# Comparative Outcomes of Meropenem–Vaborbactam vs. Ceftazidime–Avibactam Among Adults Hospitalized with an Infectious Syndrome in the US, 2019–2021

**DOI:** 10.3390/antibiotics14010029

**Published:** 2025-01-03

**Authors:** Marya D. Zilberberg, Brian H. Nathanson, Mark A. Redell, Kate Sulham, Andrew F. Shorr

**Affiliations:** 1 EviMed Research Group, LLC, Goshen, MA 01032, USA; 2 OptiStatim, LLC, Longmeadow, MA 01116, USA; brian.h.nathanson@att.net; 3 Melinta Therapeutics, Parsippany, NJ 07054, USA; mredell@melinta.com (M.A.R.); ksulham@melinta.com (K.S.); 4 Washington Hospital Center, Washington, DC 20010, USA; andrew.shorr@gmail.com

**Keywords:** comparative outcomes, multidrug resistance, hospitalization, outcomes, epidemiology

## Abstract

**Background/Objectives**: Meropenem–vaborbactam (MEV) and ceftazidime–avibactam (CZA) are active against “urgent threat” pathogens like carbapenem-resistant Enterobacterales (CRE). However, few studies have compared outcomes between them. **Methods**: To explore comparative outcomes of MEV vs. CZA, we conducted a multicenter retrospective cohort study of all adult hospitalized patients with a serious infection (sepsis, urinary tract infection [UTI], complicated intraabdominal [cIAI] infection, or pneumonia) within the PINC AI Database, 2019–2021. Descriptive statistics compared the two groups along demographic and clinical characteristics, and multiple regression derived adjusted outcomes. **Results**: Among 1,989,765 patients who met enrollment criteria, 455 received MEV and 2320 CZA. Compared to CZA, patients on MEV were more commonly Caucasian (68.1% vs. 63.6%, *p* = 0.032) or Hispanic (21.8% vs. 12.8%, *p* < 0.001). Their mean [SD] Charlson comorbidity scores did not differ (3.6 [2.5] vs. 3.5 [2.5], *p* = 0.403). The most common index infection in both groups was pneumonia, though it was less prevalent in the MEV- than the CZA-treated group (48.1% vs. 56.8%, *p* = 0.001). Fewer than one-third of all patients received the respective drug within 2 days of the onset of the index infection (30.6% MEV vs. 33.0% CZA, *p* = 0.313). Fewer patients on MEV than CZA required mechanical ventilation (35.0% vs. 41.4%, *p* = 0.010). MEV treatment was associated with lower adjusted mortality (17.0% [95% CI 13.6%, 20.3%] vs. 20.6% [95% CI 19.0%, 22.2%], *p* = 0.048) relative to CZA. **Conclusions**: In this cohort of hospitalized patients treated with either MEV or CZA for their infectious syndrome, MEV was associated with lower adjusted hospital mortality, although the confidence intervals around the values overlapped.

## 1. Introduction

Antimicrobial resistance remains a major issue in US hospitals. Carbapenem-resistant (CR) pathogens, though still relatively rare, are increasing in prevalence and present a substantial treatment challenge, as few therapeutic options exist to treat these infections effectively. In particular, carbapenem-resistant Enterobacterales (CRE) have been identified by the Centers for Disease Control and Prevention as an urgent public health threat [1]. According to the report, there are over 13,000 cases of CRE annually in US hospitals, associated with more than 1000 deaths and estimated attributable costs of $130 million [1].

Historically, CR pathogens have been treated with polymyxin-based regimens [2,3,4]. However, these agents are associated with multiple complications, most prominent of which is acute kidney injury [5,6,7]. Though some advocate for the use of select older agents used in combination for the treatment of CRE infection, newer therapeutics now exist. The pairing of established beta-lactams (BLs) with novel beta-lactamase inhibitors (BLIs), designed to enhance in vitro potency against CRE, has led to newer therapies effective against CRE, such as meropenem–vaborbactam (MEV) and ceftazidime–avibactam (CZA). The trials that led to the approval of such antibiotics generally did not compare any of these newly developed BL/BLIs combination drugs against each other and, as such, do not provide insight into whether clinically significant differences exist between these agents. Recent guidance from the Infectious Diseases Society of America underscores this fact by noting that their recommendations for the treatment of select CR infections are based mainly on small clinical trials and expert opinion [8]. Even today, there remains a paucity of generalizable data on comparative outcomes of these agents [9]. In other words, clinicians lack evidence from robust comparative outcomes or pharmacoepidemiology analyses to guide their decisions about how to choose the newer BL/BLIs.

In the current multicenter retrospective cohort study, we examine the epidemiology, treatment, and outcomes of serious infections treated with either MEV or CZA in US acute care hospitals in order to address the evidence gap related to comparative treatment outcomes of those antimicrobials.

## 2. Results

Among 1,989,765 patients who met enrollment criteria, 2775 were treated with either MEV or CZA, of whom 455 (16.4%) received MEV and 2320 (83.6%) CZA. MEV and CZA patients did not differ significantly in age (mean [SD] 62.2 [15.2] vs. 60.8 [16.0] years, *p* = 0.102) (Table 1). Compared to those on CZA, patients on MEV were more commonly Caucasian (68.1% vs. 63.6%, *p* = 0.032) or Hispanic (21.8% vs. 12.8%, *p* < 0.001). They were also more likely to suffer from diabetes and from liver disease, but less likely to have chronic pulmonary disease (Table S8). Overall chronic disease burden, however, did not differ between the groups (mean [SD] Charlson comorbidity score 3.6 [2.5] vs. 3.5 [2.5], *p* = 0.403) (Table 1). MEV was more commonly administered in hospitals in the South (57.4% vs. 45.3%, *p* < 0.001) and less so in teaching institutions (52.5% vs. 60.0%, *p* = 0.003).

The most common index infection in both groups was pneumonia, though it was less prevalent in the MEV- than the CZA-treated group (48.1% vs. 56.8%, *p* = 0.001, Table 2). Similarly, fewer patients in the MEV than in CZA group required mechanical ventilation (35.0% vs. 41.4%, *p* = 0.010). The prevalence of severe sepsis, septic shock, and vasopressor administration did not differ between the groups (Table 2). In contrast, more patients in the MEV than CZA group required dialysis (13.9% vs. 8.6%, *p* < 0.001). With the exception of antipseudomonal carbapenems (1.5% vs. 5.1%, *p* = 0.001) and antipseudomonal cephalosporins (13.6% vs. 9.1%, *p* = 0.003), all other classes of antimicrobials examined were equally likely to be administered during the hospitalization prior to the onset of the index infection of interest (Table S8). In both groups, fewer than one-third of all patients received a BL/BLI within 2 days of the onset of the index infection (30.6% MEV vs. 33.0% CZA, *p* = 0.313). The median [IQR] time to instituting these drugs from the index infection onset was 6 [1, 17] days for MEV vs. 5 days [1, 18] for CZA (*p* = 0.945), and the mean (SD) duration of administration was 7.0 (5.1) and 7.1 (5.4) days, respectively (Table S8).

With the exception of post-infection onset LOS and hospital costs, all other unadjusted outcomes were similar in the two groups (Table 3). From the inverse probability weighting models adjusting for multiple confounders, MEV treatment was associated with lower mortality (16.5%, 95% CI 13.2%, 19.7% vs. 20.6%, 95% CI 19.0%, 22.3%, *p* = 0.021) and a shorter post-infection onset ICU LOS (19.6, 95% CI 17.1, 22.1 vs. 22.4, 95% CI 21.1, 23.8 days, *p* = 0.042) relative to CZA (Table 4). We further observed a trend toward MEV-treated (vs. CZA-treated) patients having a reduced risk for ICU admission, and a trend toward lower risk for incident *C. difficile* infection, though neither met the prespecified alpha for statistical significance (Table 4).

## 3. Discussion

We demonstrate that after several years of being commercially available, either MEV or CZA is used in only a small minority of hospital-based infectious syndromes. Additionally, few patients receive either agent empirically as evidenced by late post-infection initiation of both. It is likely that both agents are used only when the results of cultures return and confirm the presence of a resistant pathogen. Furthermore, although the comparator groups in our analysis are relatively similar in terms of multiple characteristics and processes of care, and while this difference was not significant in the unadjusted estimates, MEV administration was associated with a significantly reduced hospital mortality and shorter ICU LOS after inverse probability weighting. Nonetheless, the confidence intervals for both of these values overlapped. Additionally, we observed trends toward a lower risk of incident CDI and a trend toward lower need for an ICU admission.

CR infections have been on the rise worldwide over the last decade. Though these organisms can develop resistance via multiple potential mechanisms, the production of carbapenemases represents the most common one for CRE [10,11]. At the same time, the development of new antibiotics to address these infections has lagged in comparison to changing and evolving epidemiology. MEV and CZA comprise two of the newer agents aimed at CRE infections, and data suggest they improve outcomes relative to older options. For example, a recent systematic review and meta-analysis of risk factors for mortality associated with CRE noted that while older drug combination regimens appear superior to older monotherapy, treatment with CZA reduced mortality even further [12]. While MEV was not examined in that review, multiple other comparisons have confirmed MEV-associated improved outcomes relative to older regimens [13]. On the one hand, the fact that only a small minority of patients with serious infectious syndromes receive either CZA or MEV is reassuring as it dampens concerns of overuse and a downstream loss of efficacy. On the other, this finding confirms a prior analysis by Clancy et al., which reported underuse of these agents in the settings where they would be considered appropriate first-line treatment [14]. It raises questions as to what prevents such potential appropriate use and how earlier and more appropriate utilization of these agents might impact patient outcomes.

Our study builds on prior investigations that compared the outcomes of MEV vs. CZA, though ours represents the largest cohort evaluated to date. For example, a retrospective cohort study by Ackley and colleagues found that among 131 patients with CRE infection, the treatment groups were quite similar in terms of baseline characteristics and outcomes, save for a higher prevalence in the CZA group of combination therapy [9]. Notably, these authors observed trends with MEV treatment toward a lower unadjusted 30-day mortality (11.5% MEV vs. 19.1% CZA, *p* = 0.57) and reduced median ICU LOS (12.0 [5.0, 22.0] MEV vs. 15.0 [5.0, 32.0] CZA, *p* = 0.53), though the statistical significance threshold was not met in either of these outcomes possibly due to a small sample size. Although different from the Ackley investigation along several lines, our study confirms the associated survival advantage as well as a trend toward the ICU LOS reduction among patients receiving MEV, in addition to a numerically lower risk of needing an ICU admission altogether when adjusted for potential confounders. While our study did not aim to examine the reasons for these potential differences, some may relate to the higher potency of MEV against *Klebsiella pneumoniae* carbapenemase (KPC) enzymes, a common mechanism for carbapenem resistance among Enterobacterales [10,11]. This is borne out by studies showing a high probability of MEV attaining PK-PD targets against Enterobacterales [15]. An additional factor may be the observed reduction in propensity for emergence of resistance against MEV [16].

Recent meta-analyses or systematic reviews have been reported on the efficacy and safety of MEV and CZA for treating serious infections due to Gram-negative pathogens. A meta-analysis and systematic review compared MEV versus best available therapy (BAT) for treating carbapenem-resistant Enterobacterales (Bucataru et al., 2024 [17]). BAT included mono/combination therapy with polymyxins, carbapenems, aminoglycosides, colistin, tigecycline, ceftazidime–avibactam, or piperacillin–tazobactam. No significant difference in clinical and microbiological response was found with MEV versus comparators, but 28-day all-cause mortality and the incidence of renal-related AEs were lower with MEV. In a systematic review of newer drugs approved for treating cUTI, MEV had the highest cure rate at 98.4%, followed by piperacillin–tazobactam at 94%, and CZA at 87.5% (Sivanandy et al., 2024 [18]). Another systematic review and meta-analysis compared the effectiveness of CZA in adults with bacteremia or nosocomial pneumonia due to carbapenem-resistant Enterobacterales or multi-drug resistant *Pseudomonas aeruginosa* (Shields et al., 2024 [19]). All-cause mortality and clinical cure rates were lower with CZA vs. comparators for patients with bacteremia and clinical cure rates were lower for nosocomial pneumonia. CZA was compared vs. polymyxin among patients with infections due to carbapenem-resistant Enterobacterales (Chen et al., 2024 [20]). CZA was associated with significantly lower 30-day mortality, higher clinical cure rate, and higher microbial clearance compared with polymyxin-based therapy.

An additional hypothesis-raising finding in our analysis is the association of MEV treatment with a lower incidence of CDI, a deadly and costly hospital-acquired complication that is under close scrutiny by policymakers and quality organizations, and is designated as an urgent threat by the CDC. Although prior evidence suggests that carbapenems and cephalosporins are equally likely to cause CDI, our finding merits further confirmation or refutation in future analyses.

Our study has a number of strengths and limitations. As a large multicenter cohort, it is representative of US institutions and thus has broad generalizability. The chief limitation of our study is the administrative nature of the database, which predisposes our cohort to misclassification vis-à-vis infectious syndromes. While this is the case, we maximized the specificity of our case definition by requiring multiple days of specific antimicrobials to be administered in the setting of the diagnostic algorithms. Although these algorithms have not been clinically validated, many similar studies within this dataset and other administrative databases have used an identical approach [21,22]. Additionally, we lacked the results of microbiologic cultures and therefore were unable to examine the impact of antimicrobial resistance on outcomes. Although a subset of the Premier dataset includes information about microbiologic cultures, to limit the current study to only patients with culture confirmation would severely reduce the sample size and thus undermine our aim to explore pharmacoepidemiology and outcomes. Other investigators have successfully used administrative databases for similar endeavors under the presumption that agents like MEV and CZA are used for cases of Gram-negative carbapenem resistance according to stewardship recommendations [21,23]. Another potential limitation of our study is that we did not examine combination treatments. Indeed, in the cohort described by Ackley and colleagues, more patients on CZA than MEV required combination therapy. Despite this, their findings showed the same directionality as ours, lending our results face validity.

Furthermore, as with any retrospective cohort, our study may be susceptible to selection bias. We have attempted to address this concern by setting a priori enrollment criteria and definitions for exposures and outcomes. Diminishing some of the worry over selection bias we focused on outcomes that are minimally susceptible to misclassification. However, excluding patients who either did not survive or were discharged prior to day three of antibiotics may have introduced an immortal time bias. Therefore, caution should be exercised in that our findings may not apply to those either extremely ill at the onset of infection with no hope of surviving, or those who are not ill enough to require a prolonged hospitalization. Although confounding is also a potential issue in observational studies, we sought to minimize it by adjusting for it in regression models. At the same time, residual confounding may remain. Even if this is the case, and even if the differences between CZA and MEV that we have identified are overestimates, a conclusive randomized controlled comparative outcomes trial is unlikely to ever take place given the paucity of the pathogens of interest. Therefore, an observational analysis may be the only viable way to address this important question. At the very least, we have shown that MEV and CZA achieve comparable outcomes, and the decision on which drug to utilize may come down to patient or microbiologic characteristics, cost-effectiveness, and clinical experience [14,24].

## 4. Methods

### 4.1. Ethics Statement

Because this study used fully deidentified administrative data, it was exempt from ethics review under US 45 CFR 46.101(b)4 [25].

### 4.2. Study Design and Patient Population

We conducted a multicenter retrospective cohort study of hospitalized patients with a serious infection, either community- or hospital-acquired, including urinary tract infection (UTI), pneumonia, sepsis, or complicated intra-abdominal infection (cIAI), and who at any point in their index hospitalization received MEV or CZA. The case identification approach relied on ICD-10 codes and pharmacy data (Tables S1–S4). Although we did not have access to microbiology data, as done by other investigators, we hypothesized that both MEV and CZA are used exclusively in the setting of carbapenem resistant Gram-negative pathogens to comport with stewardship recommendations [23,26]. Among these syndromes, we created four hierarchically determined mutually exclusive groups, with definitional preference given to the ICD-10 code associated with the hospitalization. That is, if a patient had evidence of multiple concurrent organ-specific infectious syndromes, they were classified according to the following hierarchy: pneumonia > cIAI > UTI. In the absence of an organ-specific infection, they were classified under sepsis. We only included a patient’s first infection meeting inclusion criteria.

Patients were included if they were adults (age ≥ 18 years) hospitalized with UTI, pneumonia, sepsis, or cIAI who received suitable antimicrobials for a serious hospital-based infection (Table S5) for 3 or more consecutive days. Those under 18 years of age, without one of the predefined infectious syndromes, no MEV or CZA treatment, and those transferred from another acute care facility were excluded.

### 4.3. Data Source

We derived the cohort from PINC AI (formerly Premier Healthcare Database), an electronic laboratory, pharmacy, and billing data repository, for years 2019 through 2021. The database contains inpatient records of 13 million hospital discharges annually, representing ~25% of all hospitalizations nationwide in approximately 900 hospitals. In addition to the standard information contained in typical hospital claims (those derived from the Uniform Billing—04 form, UB-04), such as patient age, gender, race/ethnicity, principal and secondary diagnoses, and procedures, the database contains a date-stamped log of all items and services charged to the patient or their insurer, including all medications, laboratory tests, and diagnostic and therapeutic services. PINC AI assigns each patient a unique identifier, so that previous and subsequent admissions to the same hospital, along with principal and secondary diagnoses and procedure codes, can be readily ascertained. The database has been described in detail previously [21,22].

### 4.4. Baseline Measures

In addition to infection classification, patient factors examined included history of exposure to antibiotics within 90 days prior to the index admission, antibiotic exposure during the index hospitalization prior to the onset of the infection in question, demographic variables, and comorbid conditions (Table S6). A Charlson comorbidity score was computed as a measure of the burden of chronic illness, while ICU admission, mechanical ventilation (MV), presence of severe sepsis or septic shock, need for acute dialysis, and vasopressor use were markers of acute disease severity. We also explored hospital structural characteristics (e.g., size, teaching status, urbanicity).

### 4.5. Hospital and Process of Care Variables

We examined a number of important hospital and processes of care variables relative to start of hospitalization or the infection onset. Infection onset was identified by the first day of administration of one of the suitable antimicrobials for a serious hospital-based infection (Table S7). The variables included the following: patient type (medical or surgical, as assigned in the patient record); illness severity (ICU and time to ICU admission relative to date of infection onset among those requiring any ICU); endotracheal intubation/mechanical ventilation (MV) and time to MV relative to date of infection onset among those requiring any MV (ICD-10 code 5A1945Z or 5A1955Z); vasopressors and time to vasopressors relative to date of infection onset among those requiring any vasopressors; dialysis and time to dialysis relative to date of infection onset among those requiring any dialysis; severe sepsis (ICD-10 R6520) or septic shock (ICD-10 R6521) at any time during index hospitalization; and infection type. Additional pertinent variables examined were antibiotics administered prior to the onset of infection, antibiotics administered following index infection treatment, and whether a complete course of antimicrobial therapy was administered (defined as evidence of treatment for >/= 5 consecutive days from infection onset, or a discharge from the hospital within this time frame).

### 4.6. Outcome Variables

For all analyses, the outcome variables were as follows:

Primary outcome: Hospital mortality

Secondary outcomes:

1. Hospital length of stay (LOS, days)
  - Overall
  - Post-infection onset
2. ICU LOS (days)
  - Overall
  - Post-infection onset
3. Duration of MV (days)
  - Overall
  - Post-infection onset
4. Hospital costs ($)
5. 30- and 90-day readmission rates among survivors
6. Development of *C. difficile* infection (CDI), as evidenced by the start of oral metronidazole or oral vancomycin or fidaxomicin treatment between infection day 3 and discharge from the hospital, whichever comes first.

### 4.7. Statistical Analyses

We used standard descriptive statistics to compare patient groups treated with MEV vs. CZA across demographics, comorbidities, hospital characteristics, and processes, as well as hospital outcomes. Continuous variables are reported as means with standard deviations when normally distributed and/or as medians with 25th and 75th percentiles when the distributions are skewed. Differences between mean values were tested using the Student’s *t*-test, while those between medians were examined using the Mann–Whitney U test. Categorical data are summarized as proportions. The Chi-square test or Fisher’s exact test for cell counts < 4 was used to examine inter-group differences. We used an inverse probability weighting approach to derive propensity score-adjusted outcome comparisons between the two groups (Table S8). Here, a propensity score is derived based on logistic regression with MEV vs. CZA as the outcome variable and clinically plausible patient characteristics known before infection onset as independent variables. Rather than match on patients’ propensities (probabilities) of receipt of MEV vs. CZA, the baseline characteristics are balanced by taking the inverse of the propensity (i.e., 1/(propensity score) for those receiving CZA and 1/(1 − propensity score) for those receiving MEV) for each patient to create weights. These weights are then used to create a “pseudo” population of MEV and CZA patients that balances potentially confounding patient characteristics between both treatment groups [27,28]. The inverse probability weighting approach generally uses all or almost all patients to create the balanced pseudo-population. Consequently, the analysis has more statistical power than simple propensity matching approaches, where often patients with very high or low propensities are not matched, and therefore not analyzed. Statistical significance was set at *p* < 0.05. All analyses were done using Stata/MP 18.0 for Windows software (StataCorp LLC, College Station, TX, USA).

## 5. Conclusions

In summary, both CZA and MEV appear to be administered infrequently in US hospitals. However, when used, their adjusted hospital outcomes are comparable, with MEV exhibiting potentially lower mortality and shorter ICU LOS, as well as a trend toward lower ICU admission and CDI incidence than CZA. Future studies are needed to examine this relationship of MEV with a reduced risk of CDI development. Additional work in the cost-effectiveness arena could clarify use of MEV vs. CZA in the setting of presumed or proven CRE infection.

## Figures and Tables

**Table 1:** Baseline characteristics.

	MEV	%	CZA	%	*p*-Value
	N = 455		N = 2320		
Mean age, years (SD)	62.2 (15.2)		60.8 (16)		0.102
Gender: male	255	56.04%	1406	60.60%	0.070
Race					
White	310	68.13%	1476	63.62%	0.032
Black	87	19.12%	448	19.31%	
Asian	14	3.08%	68	2.93%	
Other	26	5.71%	245	10.56%	
Unknown	18	3.96%	83	3.58%	
Hispanic	99	21.76%	296	12.76%	<0.001
Admission Source					
Non-healthcare facility (including from home)	380	83.52%	1881	81.08%	0.013
Clinic	50	10.99%	219	9.44%	
Transfer from ECF	25	5.49%	199	8.58%	
Transfer from another non-acute care facility/Other	0	0.00%	21	0.91%	
Admission Type					
Surgical	207	45.49%	1045	45.04%	0.86
Medical	248	54.51%	1275	54.96%	
Do Not Resuscitate Order					
Present on admission	51	11.21%	249	10.73%	0.765
Any time	125	27.47%	698	30.09%	0.264
COVID (incident or prevalent)	91	20.00%	433	18.66%	0.505
Charlson Comorbidity Score					
Mean (SD)	3.6 (2.5)		3.5 (2.5)		0.403
Median [IQR]	3 [2, 5]		3 [2, 5]		0.335
Hospital Characteristics					
Census region					
Midwest	89	19.56%	469	20.22%	<0.001
Northeast	60	13.19%	483	20.82%	
South	261	57.36%	1051	45.30%	
West	45	9.89%	317	13.66%	
Number of Beds					
<100	11	2.42%	45	1.94%	<0.001
100 to 199	37	8.13%	247	10.65%	
200 to 299	93	20.44%	311	13.41%	
300 to 399	59	12.97%	435	18.75%	
400 to 499	61	13.41%	267	11.51%	
500+	194	42.64%	1015	43.75%	
Teaching	239	52.53%	1391	59.96%	0.003
Urban	407	89.45%	2117	91.25%	0.221

MEV = meropenem–vaborbactam; CZA = ceftazidime–avibactam; COVID = coronavirus disease of 2019; SD = standard deviation; ECF = extended care facility; IQR = interquartile range.

**Table 2:** Infection characteristics.

	MEV	%	CZA	%	*p*-Value
	N = 455		N = 2320		
Infection Source					
UTI	89	19.56%	327	14.09%	0.003
Pneumonia	219	48.13%	1317	56.77%	0.001
Sepsis	126	27.69%	584	25.17%	0.260
cIAI	21	4.62%	92	3.97%	0.521
Time to Infection (days)					
UTI					
Mean (SD)	2.8 (4.2)		2.0 (2.1)		0.009
Median [IQR]	2 [1, 2]		1 [1, 2]		0.013
Pneumonia					
Mean (SD)	3.2 (4.5)		3.9 (13.5)		0.480
Median [IQR]	1 [1, 3]		1 [1, 3]		0.658
Sepsis					
Mean (SD)	2.7 (3)		2.7 (4.1)		0.959
Median [IQR]	1 [1, 3]		1 [1, 2]		0.404
cIAI					
Mean (SD)	3.4 (3.2)		3.3 (3.6)		0.888
Median [IQR]	2 [1, 5]		2 [1, 3]		0.832
Antibiotics within 90 days prior to admission	29	6.37%	137	5.91%	0.700
Illness Severity Measures by Day 2 from Infection Onset					
ICU admission	217	47.69%	1181	50.91%	0.210
Time to ICU Admission (days)					
Mean (SD)	4.5 (6.8)		4.6 (14.1)		0.856
Median [IQR]	1 [1, 5]		1 [1, 4]		0.248
Mechanical ventilation	159	34.95%	961	41.42%	0.010
Time from Hospitalization to MV (d)					
Mean (SD)	7.3 (10.6)		5.5 (9.1)		0.009
Median [IQR]	3 [1, 9]		2 [1, 7]		0.002
Time from Infection Onset to MV (days)					
Mean (SD)	4.8 (10.4)		3.0 (7.3)		0.001
Median [IQR]	0 [0, 5]		0 [0, 3]		0.013
Vasopressors	155	34.07%	818	35.26%	0.626
Time to Vasopressors (days)					
Mean (SD)	7 (9.5)		6.6 (10.7)		0.573
Median [IQR]	3 [1, 10]		3 [1, 8]		0.203
Dialysis	63	13.85%	200	8.62%	0.001
Time to Dialysis (days)					
Mean (SD)	11.6 (18.1)		14.2 (25.7)		0.293
Median [IQR]	4 [2, 14.5]		7 [2, 18]		0.040
Severe sepsis	45	9.89%	269	11.59%	0.294
Septic shock	205	45.05%	1017	43.84%	0.632

MEV = meropenem–vaborbactam; CZA = ceftazidime–avibactam; UTI = urinary tract infection; cIAI = complicated intra-abdominal infection; SD = standard deviation; IQR = interquartile range; ICU = intensive care unit; MV = mechanical ventilation.

**Table 3:** Unadjusted hospital outcomes.

	MEV	%	CZA	%	*p*-Value
	N = 455		N = 2320		
Hospital mortality, %	95	20.88%	474	20.43%	0.829
30-day readmission, %	67	14.73%	351	15.13%	0.826
90-day readmission, %	113	24.84%	582	25.09%	0.910
Hospital LOS, days					
Mean (SD)	29.7 (31.2)		31.9 (38.1)		0.238
Median [IQR]	20 [11, 37]		21 [10, 41]		0.929
Hospital costs, $					
Mean (SD)	112,762 (148,068)		136,789 (207,819)		0.019
Median [IQR]	65,044 [32,778, 125,856]		69,815 [31,199, 157,554]		0.109
Post-infection Onset Hospital Costs, $					
Mean (SD)	103,585 (137,262)		127,696 (198,537)		0.013
Median [IQR]	60,247 [29,326, 116,172]		62,766 [28,131, 147,525]		0.120
Post-infection Onset Hospital LOS, (days)					
Mean (SD)	26.7 (30.1)		28.6 (35.9)		0.273
Median [IQR]	18 [9, 33]		17 [9, 36.5]		0.827
ICU admission, %	294	64.62%	1525	65.73%	0.647
ICU LOS, days					
Mean (SD)	20.3 (20.8)		23.3 (28.5)		0.091
Median [IQR]	15 [6, 28]		15 [5, 31]		0.732
Post-infection Onset ICU LOS, days					
Mean (SD)	18.7 (20.1)		21.4 (27.9)		0.118
Median [IQR]	14 [5, 26]		13 [4, 29]		0.952
MV, %	241	52.97%	1328	57.24%	0.093
MV Duration, days					
Mean (SD)	22.5 (22)		26 (30.1)		0.089
Median [IQR]	15 [7, 31]		18 [8, 35]		0.110
Post-infection Onset MV Duration, days					
Mean (SD)	21.3 (21.3)		24.4 (29.8)		0.119
Median [IQR]	14 [7, 30]		17 [7, 32]		0.232
Incident AKI, %	245	53.85%	1313	56.59%	0.280
Incident AKI not POA, %	80	17.58%	449	19.35%	0.379
Incident *C. difficile*, %	55	12.09%	331	14.27%	0.219

AKI = acute kidney injury; CZA = ceftazidime–avibactam; ICU = intensive care unit; IQR = interquartile range; LOS = length of stay; MEV = meropenem–vaborbactam; MV = mechanical ventilation; POA = present on admission; SD = standard deviation.

**Table 4:** Adjusted outcomes *.

Outcome	MEV	CZA	*p*-Value
Hospital mortality, % (95% CI)	16.5% (13.2%, 19.7%)	20.6% (19.0%, 22.3%)	0.021
30-day readmission, % (95% CI)	16.7% (13.0%, 20.5%)	18.8% (17.1%, 20.6%)	0.325
90-day readmission, % (95% CI)	30.6% (25.5%, 35.7%)	31.4% (29.3%, 33.5%)	0.766
Post-infection onset hospital costs (95% CI)	$121,232 (103,666, 138,798)	$126,854 (118,469, 134,524)	0.564
Post-infection onset hospital LOS, days (95% CI)	29.5 (25.9, 33.1)	28.5 (27.1, 29.9)	0.604
Post-infection onset hospital LOS among survivors, days (95% CI)	26.9 (22.8, 31.1)	26.3 (24.8, 27.8)	0.775
Post-infection onset ICU admission (on or after infection onset day), % (95% CI)	40.2% (35.6%, 44.9%)	43.9% (41.9%, 45.9%)	0.135
Post-infection onset ICU admission (after infection onset day), % (95% CI)	16.9% (13.7%, 20.2%)	18.4% (16.8%, 19.9%)	0.405
Post-infection onset ICU LOS, days (95% CI)	19.6 (17.1, 22.1)	22.4 (21.1, 23.8)	0.042
Post-infection onset MV (on or after infection onset day), % (95% CI)	38.7% (34.2%, 43.1%)	40.8% (38.9%, 42.8%)	0.357
Post-infection onset MV (after infection onset day), % (95% CI)	20.2% (17.0%, 23.5%)	22.1% (20.4%, 23.7%)	0.289
Post-infection onset MV duration, days (95% CI)	25.6 (22.4, 28.7)	24.7 (23.1, 26.3)	0.634
Incident *C. difficile*, % (95% CI)	11.5% (8.5%, 14.6%)	14.2% (12.8%, 15.6%)	0.116

* Inverse probability weighting used to derive adjusted outcomes, MEV = meropenem–vaborbactam; CZA = ceftazidime–avibactam; CI = confidence interval; LOS = length of stay; ICU = intensive care unit; MV = mechanical ventilation.

## Data Availability

The data underlying the study derive from a third party deidentified database, Premier Healthcare database available from Premier Inc./PINC AI. The authors did not have any special access privileges that are not available to any other researchers wishing to purchase access to the database. Any researcher is able to obtain access to these data by contacting Premier as follows: Jim Sianis, BS, PharmD, MBA, Senior Director, Real World Evidence, Applied Sciences I Premier Inc. james_sianis@premierInc.com.

## Supplementary Materials

The following supporting information can be downloaded at: https://www.mdpi.com/article/10.3390/antibiotics14010029/s1.
