# Supplementary material for: Comparative Outcomes of Meropenem–Vaborbactam vs. Ceftazidime–Avibactam Among Adults Hospitalized with an Infectious Syndrome in the US, 2019–2021
Source: Antibiotics (Basel). 2025 Jan 3;14(1):29. doi: 10.3390/antibiotics14010029 (PMC11762528; doi:10.3390/antibiotics14010029)
Supplement: Supplementary file 1 [file antibiotics-14-00029-s001.zip › Supplemental Table S1.pdf]

Supplemental Table S1. Pneumonia ICD-10 codes

ICD-10 code	Description
A481	Legionnaires' disease
J13	Pneumonia due to Streptococcus pneumoniae
J14	Pneumonia due to Hemophilus influenzae
J1520	Pneumonia due to staphylococcus, unspecified
J15211	Pneumonia due to methicillin susceptible Staphylococcus aureus
J15212	Pneumonia due to methicillin resistant Staphylococcus aureus
J1529	Pneumonia due to other staphylococcus
J153	Pneumonia due to streptococcus, group B
J154	Pneumonia due to other streptococci
J157	Pneumonia due to Mycoplasma pneumoniae
J158	Pneumonia due to other specified bacteria
J159	Unspecified bacterial pneumonia
J160	Chlamydial pneumonia
J168	Pneumonia due to other specified infectious organisms
J17	Pneumonia in diseases classified elsewhere
J180	Bronchopneumonia, unspecified organism
J181	Lobar pneumonia, unspecified organism
J182	Hypostatic pneumonia, unspecified organism
J188	Other pneumonia, unspecified organism
J189	Pneumonia, unspecified organism
J1000	Influenza due to other identified influenza virus with unspecified type of pneumonia
J1008	Influenza due to other identified influenza virus with other specified pneumonia
J1100	Influenza due to unidentified influenza virus with unspecified type of pneumonia
J1108	Influenza due to unidentified influenza virus with specified pneumonia
J120	Adenoviral pneumonia
J121	Respiratory syncytial virus pneumonia
J122	Parainfluenza virus pneumonia
J123	Human metapneumovirus pneumonia
J1281	Pneumonia due to SARS-associated coronavirus
J1289	Other viral pneumonia
J129	Viral pneumonia, unspecified
J150	Pneumonia due to Klebsiella pneumoniae
J151	Pneumonia due to Pseudomonas
J155	Pneumonia due to Escherichia coli
J156	Pneumonia due to other aerobic Gram-negative bacteria

J69.0	Pneumonitis due to inhalation of food and vomit
J69.1	Pneumonitis due to inhalation of oils and essences
J69.8	Pneumonitis due to inhalation of other solids and liquids
J95.851	VAP
