# Supplementary material for: Comparative Outcomes of Meropenem–Vaborbactam vs. Ceftazidime–Avibactam Among Adults Hospitalized with an Infectious Syndrome in the US, 2019–2021
Source: Antibiotics (Basel). 2025 Jan 3;14(1):29. doi: 10.3390/antibiotics14010029 (PMC11762528; doi:10.3390/antibiotics14010029)
Supplement: Supplementary file 1 [file antibiotics-14-00029-s001.zip › Supplemental Table S2.pdf]

Supplemental Table S2. Sepsis ICD-10 codes

ICD-10 code	Description
A400	Sepsis due to streptococcus, group A
A401	Sepsis due to streptococcus, group B
A403	Sepsis due to Streptococcus pneumoniae
A408	Other streptococcal sepsis
A409	Streptococcal sepsis, unspecified
A4101	Sepsis due to methicillin susceptible Staphylococcus aureus
A4102	Sepsis due to methicillin resistant Staphylococcus aureus
A411	Sepsis due to other specified staphylococcus
A412	Sepsis due to unspecified staphylococcus
A413	Sepsis due to Hemophilus influenzae
A414	Sepsis due to anaerobes
A4150	Gram-negative sepsis, unspecified
A4151	Sepsis due to Escherichia coli [E. coli]
A4152	Sepsis due to Pseudomonas
A4153	Sepsis due to Serratia
A4159	Other Gram-negative sepsis
A4181	Sepsis due to Enterococcus
A4189	Other specified sepsis
A419	Sepsis, unspecified organism
R6520	Severe sepsis without septic shock
R6521	Severe sepsis with septic shock
