# Supplementary material for: Comparative Outcomes of Meropenem–Vaborbactam vs. Ceftazidime–Avibactam Among Adults Hospitalized with an Infectious Syndrome in the US, 2019–2021
Source: Antibiotics (Basel). 2025 Jan 3;14(1):29. doi: 10.3390/antibiotics14010029 (PMC11762528; doi:10.3390/antibiotics14010029)
Supplement: Supplementary file 1 [file antibiotics-14-00029-s001.zip › Supplemental Table S3.pdf]

Supplemental table S3. UTI identification algorithm

Complicated UTI	Description	ICD-10
	Infection and inflammatory reaction due to indwelling urinary catheter	T83.511X
	Acute pyelonephritis	N10
	Chronic pyelonephritis	N11.0, N11.1
	Unspecified pyelonephritis	N12
	Pyeloureteritis cystica	N28.85
	Insertion of indwelling urinary catheter	0T9B70Z
	Replacement of indwelling urinary catheter	0T9B80Z<br>0T2BX0Z
	Irrigation of indwelling urinary catheter	3C1ZX8Z
	Fitting and adjustment of urinary devices	3E1K78Z
	Attention to other artificial opening of urinary tract	3E1K88Z<br>Z46.6<br>Z43.6
**Uncomplicated UTI**	**Description**	**ICD-10**
	Cystitis	N30.0
	Acute cystitis without hematuria	N30.00
	Acute cystitis with hematuria	N30.01
	Cystitis, unspecified	N30.9
	Cystitis, unspecified without hematuria	N30.90
	Cystitis, unspecified with hematuria	N30.91
	Nonspecific urethritis	N34.1
	Other urethritis	N34.2
	Urinary tract infection, site not specified	N39.0
