# Supplementary material for: Comparative Outcomes of Meropenem–Vaborbactam vs. Ceftazidime–Avibactam Among Adults Hospitalized with an Infectious Syndrome in the US, 2019–2021
Source: Antibiotics (Basel). 2025 Jan 3;14(1):29. doi: 10.3390/antibiotics14010029 (PMC11762528; doi:10.3390/antibiotics14010029)
Supplement: Supplementary file 1 [file antibiotics-14-00029-s001.zip › Supplemental Table S4.pdf]

Supplemental Table S4. **cIAI identification algorithm**

cIAI = at least 1 diagnosis code + at least 1 procedure code

DIAGNOSIS CODES

ICD-10 Diagnosis

K25.1	K63.0	K80.37
K25.5	K63.1	K80.4
K25.6	K63.2	K80.40
K25.2	K63.3	K80.41
K26.1	K75.0	K80.42
K26.2	K80.0	K80.43
K26.5	K80.00	K80.44
K26.6	K80.01	K80.45
K27.1	K80.1	K80.46
K27.2	K80.10	K80.47
K27.5	K80.11	K80.6
K27.6	K80.12	K80.60
K28.1	K80.13	K80.61
K28.2	K80.18	K80.62
K28.5	K80.19	K80.63
K28.6	K80.3	K80.64
K35.2	K80.30	K80.65
K35.3	K80.31	K80.66
K67	K80.32	K80.67
K65.0	K80.33	K80.8
K65.1	K80.34	K80.80
K65.3	K80.35	K80.81
K65.8	K80.36	

PROCEDURE CODES

ICD-10 Procedure

0DB40ZZ
0DB43ZZ
0DB44ZZ
0DB47ZZ
0DT40ZZ
0DT44ZZ
0DT47ZZ
0DT48ZZ
0DB60ZZ
0DB63ZZ
0DB67ZZ
0DT70ZZ
0DT74ZZ
0DT77ZZ
0DT78ZZ
0D160ZA
0D164ZA
0D168ZA
0DB60ZZ
0DB63ZZ
0DB64ZZ
0DB67ZZ
0DB68ZZ
0D160ZA
0D164ZA
0D168ZA
0DB60ZZ
0DB63ZZ
0DB64ZZ
0DB67ZZ
0DB68ZZ
0DB64Z3
0DB60ZZ
0DB63ZZ
0DB67ZZ
0D13079
0D1307A
0D1307B
0DT60ZZ
0DT64ZZ
0DT67ZZ
0DT68ZZ
0DT60ZZ
0DT64ZZ
0DT67ZZ
0DT68ZZ
0DQ60ZZ
0DQ63ZZ
0DQ64ZZ
0DQ67ZZ
0DQ68ZZ
0DQ90ZZ
0DQ93ZZ
0DQ94ZZ
0DQ97ZZ
0DQ98ZZ
0DQ60ZZ
0DQ63ZZ
0DQ64ZZ
0DQ67ZZ
0DQ68ZZ
0DQ90ZZ
0DQ93ZZ
0DQ94ZZ
0DQ97ZZ
0DQ98ZZ
0DQ60ZZ
0DQ63ZZ
0DQ64ZZ
0DQ67ZZ
0DQ68ZZ
0DB80ZZ
0DB83ZZ
0DB84ZZ
0DB87ZZ
0DB88ZZ
0DT90ZZ
0DT94ZZ
0DT97ZZ
0DT98ZZ
0DTA0ZZ
0DTA4ZZ
0DTA7ZZ
0DTA8ZZ
0DTB0ZZ
0DTB4ZZ
0DTB7ZZ
0DTB8ZZ
0DT80ZZ
0DT84ZZ
0DT87ZZ
0DT88ZZ
0DBE0ZZ
0DBE3ZZ
0DBE7ZZ
0DBE8ZZ
0DTH0ZZ
0DTH7ZZ
0DTH8ZZ
0DTF0ZZ
0DTF7ZZ
0DTF8ZZ
0DTK0ZZ
0DTL0ZZ
0DTL7ZZ
0DTL8ZZ
0DTLFZZ
0DTG0ZZ
0DTG7ZZ
0DTG8ZZ
0DTGFZZ
0DTN0ZZ
0DTN7ZZ
0DTN8ZZ
0DTNFZZ
0DBE0ZZ
0DBE3ZZ
0DBE7ZZ
0DBE8ZZ
0DBGFZZ
0DBLFZZ
0DBMFZZ
0DBNFZZ
0DTMFZZ
0DTE4ZZ
0DTE0ZZ
0DTE7ZZ
0DTE8ZZ
0D190Z9
0D190ZA
0D190ZB
0D190ZL

0D194Z9
0D194ZA
0D194ZB
0D194ZL
0D198Z9
0D198ZA
0D198ZB
0D198ZL
0D1A0ZA
0D1A0ZB
0D1A0ZH
0D1A0ZK
0D1A0ZL
0D1A0ZM
0D1A0ZN
0D1A0ZP
0D1A4ZA
0D1A4ZB
0D1A4ZH
0D1A4ZK
0D1A4ZL
0D1A4ZM
0D1A4ZN
0D1A4ZP
0D1A8ZA
0D1A8ZB
0D1A8ZH
0D1A8ZK
0D1A8ZL
0D1A8ZM
0D1A8ZN
0D1A8ZP
0D1B0ZB
0D1B0ZH
0D1B0ZK
0D1B0ZL
0D1B0ZM
0D1B0ZN
0D1B0ZP
0D1B4ZB
0D1B4ZH
0D1B4ZK
0D1B4ZL
0D1B4ZM
0D1B4ZN
0D1B4ZP
0D1B8ZB
0D1B8ZH
0D1B8ZK
0D1B8ZL
0D1B8ZM
0D1B8ZN
0D1B8ZP
0D1H0ZH
0D1H0ZK
0D1H0ZL
0D1H0ZM
0D1H0ZN
0D1H0ZP
0D1H4ZH
0D1H4ZK
0D1H4ZL
0D1H4ZM
0D1H4ZN
0D1H4ZP
0D1H8ZH
0D1H8ZK
0D1H8ZL
0D1H8ZM
0D1H8ZN
0D1H8ZP
0D1K0ZK
0D1K0ZL
0D1K0ZM
0D1K0ZN
0D1K0ZP
0D1K4ZK
0D1K4ZL
0D1K4ZM
0D1K4ZN
0D1K4ZP
0D1K8ZK
0D1K8ZL
0D1K8ZM
0D1K8ZN
0D1K8ZP
0D1L0Z4
0D1L0ZL
0D1L0ZM
0D1L0ZN
0D1L0ZP
0D1L4Z4
0D1L4ZL
0D1L4ZM
0D1L4ZN
0D1L4ZP
0D1L8ZL
0D1L8ZM
0D1L8ZN
0D1L8ZP
0D1M0Z4
0D1M0ZM
0D1M0ZN
0D1M0ZP
0D1M4Z4
0D1M4ZM
0D1M4ZN
0D1M4ZP
0D1M8ZM
0D1M8ZN
0D1M8ZP
0D1N0Z4
0D1N0ZN
0D1N0ZP
0D1N4Z4
0D1N4ZN
0D1N4ZP
0D1N8ZN
0D1N8ZP
0D190Z9
0D190ZA
0D190ZB
0D194Z9
0D194ZA
0D194ZB
0D198Z9
0D198ZA
0D198ZB
0D1A0ZA
0D1A0ZB
0D1A4ZA
0D1A4ZB
0D1A8ZA
0D1A8ZB
0D1A8ZH
0D1B0ZB
0D1B4ZB
0D1B8ZB
0D1B8ZH
0D1A0ZP
0D1A4ZP
0D1A8ZP
0D1B0ZP
0D1B4ZP
0D1B8ZP
0D1H8ZP
0D190ZL

0D194ZL
0D198ZL
0D1A0ZH
0D1A0ZK
0D1A0ZL
0D1A0ZM
0D1A0ZN
0D1A4ZH
0D1A4ZK
0D1A4ZL
0D1A4ZM
0D1A4ZN
0D1A8ZK
0D1A8ZL
0D1A8ZM
0D1A8ZN
0D1B0ZH
0D1B0ZK
0D1B0ZL
0D1B0ZM
0D1B0ZN
0D1B4ZH
0D1B4ZK
0D1B4ZL
0D1B4ZM
0D1B4ZN
0D1B8ZK
0D1B8ZL
0D1B8ZM
0D1B8ZN
0D1H0ZH
0D1H0ZK
0D1H0ZL
0D1H0ZM
0D1H0ZN
0D1H0ZP
0D1H4ZH
0D1H4ZK
0D1H4ZL
0D1H4ZM
0D1H4ZN
0D1H4ZP
0D1H8ZH
0D1H8ZK
0D1H8ZL
0D1H8ZM
0D1H8ZN
0D1K0ZK
0D1K0ZL
0D1K0ZM
0D1K0ZN
0D1K0ZP
0D1K4ZK
0D1K4ZL
0D1K4ZM
0D1K4ZN
0D1K4ZP
0D1K8ZK
0D1K8ZL
0D1K8ZM
0D1K8ZN
0D1K8ZP
0D1L0ZL
0D1L0ZM
0D1L0ZN
0D1L0ZP
0D1L4ZL
0D1L4ZM
0D1L4ZN
0D1L4ZP
0D1L8ZL
0D1L8ZM
0D1L8ZN
0D1L8ZP
0D1M0ZM
0D1M0ZN
0D1M0ZP
0D1M4ZM
0D1M4ZN
0D1M4ZP
0D1M8ZM
0D1M8ZN
0D1M8ZP
0D1N0ZN
0D1N0ZP
0D1N4ZN
0D1N4ZP
0D1N8ZN
0D1N8ZP
0D1B0ZQ
0D1B4ZQ
0D1B8ZQ
0D190Z4
0D194Z4
0D198Z4
0D1A0Z4
0D1A4Z4
0D1A8Z4
0D1B8Z4
0DB87ZZ
0DB97ZZ
0DBA7ZZ
0DBB7ZZ
0D1K0Z4
0D1K4Z4
0D1K8Z4
0D1L0Z4
0D1L4Z4
0D1L8Z4
0D1N0Z4
0D1N4Z4
0DBE0ZZ
0DBF0ZZ
0DBG0ZZ
0DBK0ZZ
0DBL0ZZ
0DBM0ZZ
0DBN0ZZ
0D1H0Z4
0D1H4Z4
0D1H8Z4
0D1K0Z4
0D1K4Z4
0D1K8Z4
0D1L0Z4
0D1L4Z4
0D1L8Z4
0D1N0Z4
0D1N4Z4
0D1N8Z4
0D1H0Z4
0D1H4Z4
0D1H8Z4
0D1K0Z4
0D1K4Z4
0D1K8Z4
0D1L0Z4
0D1L4Z4
0D1L8Z4
0D1N0Z4
0D1N4Z4
0D1N8Z4
0D1H0Z4
0D1H4Z4
0D1H8Z4
0D1K0Z4
0D1K4Z4

0D1K8Z4
0D1L0Z4
0D1L4Z4
0D1L8Z4
0D1N0Z4
0D1N4Z4
0D1N8Z4
0D1B0Z4
0D1B4Z4
0D1B8Z4
0D1B0Z4
0D1B4Z4
0D1B8Z4
0D1B0Z4
0D1B4Z4
0D1B8Z4
0D1B0Z4
0D1B4Z4
0D1B8Z4
0DQ90ZZ
0DQ93ZZ
0DQ94ZZ
0DQ97ZZ
0DQ98ZZ
0DQ90ZZ
0DQ93ZZ
0DQ94ZZ
0DQ97ZZ
0DQ98ZZ
0DQ80ZZ
0DQ83ZZ
0DQ84ZZ
0DQ87ZZ
0DQ88ZZ
0DQA0ZZ
0DQA3ZZ
0DQA4ZZ
0DQA7ZZ
0DQA8ZZ
0DQB0ZZ
0DQB3ZZ
0DQB4ZZ
0DQB7ZZ
0DQB8ZZ
0DQ80ZZ
0DQ80ZZ
0DQ83ZZ
0DQ83ZZ
0DQ84ZZ
0DQ84ZZ
0DQ87ZZ
0DQ87ZZ
0DQ88ZZ
0DQ88ZZ
0DQA0ZZ
0DQA3ZZ
0DQA4ZZ
0DQA7ZZ
0DQA8ZZ
0DQB0ZZ
0DQB0ZZ
0DQB3ZZ
0DQB3ZZ
0DQB4ZZ
0DQB4ZZ
0DQB7ZZ
0DQB7ZZ
0DQB8ZZ
0DQB8ZZ
0DQE0ZZ
0DQE3ZZ
0DQE4ZZ
0DQE7ZZ
0DQE8ZZ
0DQN0ZZ
0DQN3ZZ
0DQN4ZZ
0DQN7ZZ
0DQN8ZZ
0DQP0ZZ
0DQP3ZZ
0DQP4ZZ
0DQP7ZZ
0DQP8ZZ
0HQ6XZZ
0HQ7XZZ
0DQE0ZZ
0DQE3ZZ
0DQE4ZZ
0DQE7ZZ
0DQE8ZZ
0DQH0ZZ
0DQH3ZZ
0DQH4ZZ
0DQH7ZZ
0DQH8ZZ
0DQK0ZZ
0DQK3ZZ
0DQK4ZZ
0DQK7ZZ
0DQK8ZZ
0DQN0ZZ
0DQN3ZZ
0DQN4ZZ
0DQN7ZZ
0DQN8ZZ
0DQE0ZZ
0DQE3ZZ
0DQE4ZZ
0DQE7ZZ
0DQE8ZZ
0DQH0ZZ
0DQH3ZZ
0DQH4ZZ
0DQH7ZZ
0DQH8ZZ
0DQN0ZZ
0DQN0ZZ
0DQN3ZZ
0DQN3ZZ
0DQN4ZZ
0DQN4ZZ
0DQN7ZZ
0DQN7ZZ
0DQN8ZZ
0DQN8ZZ
0HQ9XZZ
0DQ90ZZ
0DQ93ZZ
0DQ94ZZ
0DQ97ZZ
0DQ98ZZ
0DQE0ZZ
0DQE3ZZ
0DQE4ZZ
0DQE7ZZ
0DQE8ZZ
0DS80ZZ
0DS84ZZ
0DS87ZZ
0DS88ZZ
0DS90ZZ
0DS94ZZ
0DS97ZZ
0DS98ZZ
0DSA0ZZ
0DSA4ZZ

0DSA7ZZ	0DSE8ZZ	0DQE8ZZ
0DSA8ZZ	0DSH0ZZ	0DQ80ZZ
0DSB0ZZ	0DSH4ZZ	0DQ83ZZ
0DSB4ZZ	0DSH7ZZ	0DQ84ZZ
0DSB7ZZ	0DSH8ZZ	0DQ87ZZ
0DSB8ZZ	0DSK0ZZ	0DQ88ZZ
0DSE0ZZ	0DSK4ZZ	0DQE0ZZ
0DSE4ZZ	0DSK7ZZ	0DQE3ZZ
0DSE7ZZ	0DSK8ZZ	0DQE4ZZ
0DSE8ZZ	0DSL0ZZ	0DQE7ZZ
0DSH0ZZ	0DSL4ZZ	0DQE8ZZ
0DSH4ZZ	0DSL7ZZ	0DTJ4ZZ
0DSH7ZZ	0DSL8ZZ	0DTJ0ZZ
0DSH8ZZ	0DSM0ZZ	0DTJ7ZZ
0DSK0ZZ	0DSM4ZZ	0DTJ8ZZ
0DSK4ZZ	0DSM7ZZ	0DTJ4ZZ
0DSK7ZZ	0DSM8ZZ	0DTJ0ZZ
0DSK8ZZ	0DSN0ZZ	0DTJ7ZZ
0DSL0ZZ	0DSN4ZZ	0DTJ8ZZ
0DSL4ZZ	0DSN7ZZ	0D9J00Z
0DSL7ZZ	0DSN8ZZ	0D9J0ZZ
0DSL8ZZ	0D7N0ZZ	0D9J30Z
0DSM0ZZ	0D7N3ZZ	0D9J3ZZ
0DSM4ZZ	0D7N4ZZ	0D9J40Z
0DSM7ZZ	0D780ZZ	0D9J4ZZ
0DSM8ZZ	0D783ZZ	0D9J70Z
0DSN0ZZ	0D784ZZ	0D9J7ZZ
0DSN4ZZ	0D7E0ZZ	0D9J80Z
0DSN7ZZ	0D7E3ZZ	0D9J8ZZ
0DSN8ZZ	0D7E4ZZ	0D9J00Z
0DS80ZZ	0DQ80ZZ	0D9J0ZZ
0DS84ZZ	0DQ83ZZ	0D9J40Z
0DS87ZZ	0DQ84ZZ	0D9J4ZZ
0DS88ZZ	0DQ87ZZ	0D9J70Z
0DS90ZZ	0DQ88ZZ	0D9J7ZZ
0DS94ZZ	0DQA0ZZ	0D9J80Z
0DS97ZZ	0DQA3ZZ	0D9J8ZZ
0DS98ZZ	0DQA4ZZ	0DQJ0ZZ
0DSA0ZZ	0DQA7ZZ	0DQJ0ZZ
0DSA4ZZ	0DQA8ZZ	0DQJ3ZZ
0DSA7ZZ	0DQB0ZZ	0DQJ3ZZ
0DSA8ZZ	0DQB3ZZ	0DQJ4ZZ
0DSB0ZZ	0DQB4ZZ	0DQJ4ZZ
0DSB4ZZ	0DQB7ZZ	0DQJ7ZZ
0DSB7ZZ	0DQB8ZZ	0DQJ7ZZ
0DSB8ZZ	0DQE0ZZ	0DQJ8ZZ
0DSE0ZZ	0DQE3ZZ	0DQJ8ZZ
0DSE4ZZ	0DQE4ZZ	0HQ6XZZ
0DSE7ZZ	0DQE7ZZ	0HQ7XZZ

0DQJ0ZZ	0FQ00ZZ	0F140D5
0DQJ3ZZ	0FQ03ZZ	0F140D6
0DQJ4ZZ	0FQ04ZZ	0F140D7
0DQJ7ZZ	0FQ00ZZ	0F140Z5
0DQJ8ZZ	0FQ03ZZ	0F140Z6
0F9000Z	0FQ04ZZ	0F140Z7
0F900ZZ	0FS00ZZ	0F144D5
0FC00ZZ	0FS04ZZ	0F144D6
0FC03ZZ	0F9430Z	0F144D7
0FC04ZZ	0F940ZZ	0F144Z5
0FH00YZ	0F9400Z	0F144Z6
0FH03YZ	0FC40ZZ	0F144Z7
0FH04YZ	0FC43ZZ	0F140D3
0FP00YZ	0FC44ZZ	0F140DB
0FP03YZ	0FC48ZZ	0F140Z3
0FP04YZ	0FF40ZZ	0F140ZB
0FW00YZ	0FF43ZZ	0F144D3
0FW03YZ	0FF44ZZ	0F144DB
0FW04YZ	0FF47ZZ	0F144Z3
0F900ZX	0FH40YZ	0F144ZB
0FB00ZX	0FH43YZ	NoPCS
0FB03ZX	0FH44YZ	0F140D4
0FB04ZX	0FP40YZ	0F140Z4
0FJ03ZZ	0FP43YZ	0F144D4
0F900ZZ	0FP44YZ	0F144Z4
0F903ZZ	0FW40YZ	0F140D8
0F904ZZ	0FW43YZ	0F140D9
0FB00ZZ	0FW44YZ	0F140Z8
0FB03ZZ	0F940ZX	0F140Z9
0FB04ZZ	0F950ZX	0F144D8
0F500ZZ	0F960ZX	0F144D9
0F503ZZ	0F970ZX	0F144Z8
0F504ZZ	0F980ZX	0F144Z9
0F500ZZ	0F990ZX	0F190D3
0F500ZZ	0F9C0ZX	0F190Z3
0F503ZZ	0F9D0ZX	0F194D3
0F504ZZ	0FB40ZX	0F194Z3
0FT10ZZ	0FB50ZX	0F150D3
0FT14ZZ	0FB60ZX	0F150DB
0FT20ZZ	0FB70ZX	0F150Z3
0FT24ZZ	0FB80ZX	0F150ZB
0FT00ZZ	0FB90ZX	0F154D3
0FT04ZZ	0FBC0ZX	0F154DB
0FY00Z0	0FB40ZZ	0F154Z3
0FY00Z1	0FB43ZZ	0F154ZB
0FY00Z2	0FT40ZZ	0F160D3
0FY00Z0	0FT44ZZ	0F160DB
0FY00Z1	0FB44ZZ	0F160Z3
0FY00Z2	0FB48ZZ	0F160ZB

0F164D3	0F160Z5	0F184D6
0F164DB	0F160Z6	0F184D7
0F164Z3	0F160Z7	0F184D8
0F164ZB	0F160Z8	0F184D9
0F170D3	0F160Z9	0F184Z4
0F170DB	0F164D5	0F184Z5
0F170Z3	0F164D6	0F184Z6
0F170ZB	0F164D7	0F184Z7
0F174D3	0F164D8	0F184Z8
0F174DB	0F164D9	0F184Z9
0F174Z3	0F164Z5	0F190D4
0F174ZB	0F164Z6	0F190D5
0F180D3	0F164Z7	0F190D6
0F180DB	0F164Z8	0F190D7
0F180Z3	0F164Z9	0F190D8
0F180ZB	0F170D5	0F190D9
0F184D3	0F170D6	0F190Z4
0F184DB	0F170D7	0F190Z5
0F184Z3	0F170D8	0F190Z6
0F184ZB	0F170D9	0F190Z7
0F190DB	0F170Z5	0F190Z8
0F190ZB	0F170Z6	0F190Z9
0F194DB	0F170Z7	0F194D4
0F194ZB	0F170Z8	0F194D5
0F150D5	0F170Z9	0F194D6
0F150D6	0F174D5	0F194D7
0F150D7	0F174D6	0F194D8
0F150D8	0F174D7	0F194D9
0F150D9	0F174D8	0F194Z4
0F150Z5	0F174D9	0F194Z5
0F150Z6	0F174Z5	0F194Z6
0F150Z7	0F174Z6	0F194Z7
0F150Z8	0F174Z7	0F194Z8
0F150Z9	0F174Z8	0F194Z9
0F154D5	0F174Z9	0FC90ZZ
0F154D6	0F180D4	0FC90ZZ
0F154D7	0F180D5	0F9970Z
0F154D8	0F180D6	0FC50ZZ
0F154D9	0F180D7	0FC60ZZ
0F154Z5	0F180D8	0FC70ZZ
0F154Z6	0F180D9	0FC80ZZ
0F154Z7	0F180Z4	0FF50ZZ
0F154Z8	0F180Z5	0FF53ZZ
0F154Z9	0F180Z6	0FF54ZZ
0F160D5	0F180Z7	0FF57ZZ
0F160D6	0F180Z8	0FF60ZZ
0F160D7	0F180Z9	0FF63ZZ
0F160D8	0F184D4	0FF64ZZ
0F160D9	0F184D5	0FF67ZZ

0FF70ZZ
0FF73ZZ
0FF74ZZ
0FF77ZZ
0FF80ZZ
0FF83ZZ
0FF84ZZ
0FF87ZZ
0FF90ZZ
0FF93ZZ
0FF94ZZ
0FF97ZZ
0FFC0ZZ
0FFC3ZZ
0FFC4ZZ
0FFC7ZZ
0F9900Z
0F990ZZ
0F9940Z
0FJB0ZZ
0FJB4ZZ
0F9500Z
0F950ZZ
0F9540Z
0F954ZZ
0F9570Z
0F957ZZ
0F9580Z
0F9600Z
0F960ZZ
0F9640Z
0F964ZZ
0F9670Z
0F967ZZ
0F9680Z
0F9700Z
0F970ZZ
0F9740Z
0F974ZZ
0F9770Z
0F977ZZ
0F9780Z
0F9800Z
0F980ZZ
0F9840Z
0F984ZZ
0F9870Z
0F987ZZ
0F9880Z
0FHB0DZ
0FHB0YZ
0FHB3DZ
0FHB3YZ
0FHB4YZ
0FHB7DZ
0FJB0ZZ
0FPB0YZ
0FPB3YZ
0FPB4YZ
0FWB0YZ
0FWB3YZ
0FWB4YZ
0FWB7YZ
0FWB8YZ
0FB80ZZ
0FB83ZZ
0FB87ZZ
0FBC0ZZ
0FBC3ZZ
0FBC7ZZ
0FTC0ZZ
0FTC4ZZ
0FTC7ZZ
0FTC8ZZ
0FB90ZZ
0FB93ZZ
0FB97ZZ
0FT90ZZ
0FT94ZZ
0FT97ZZ
0FT98ZZ
0F550ZZ
0F553ZZ
0F557ZZ
0F560ZZ
0F563ZZ
0F567ZZ
0F570ZZ
0F573ZZ
0F577ZZ
0F580ZZ
0F583ZZ
0F587ZZ
0FB50ZZ
0FB53ZZ
0FB57ZZ
0FB60ZZ
0FB63ZZ
0FB67ZZ
0FB70ZZ
0FB73ZZ
0FB77ZZ
0FB80ZZ
0FB83ZZ
0FB87ZZ
0FT50ZZ
0FT54ZZ
0FT57ZZ
0FT58ZZ
0FT60ZZ
0FT64ZZ
0FT67ZZ
0FT68ZZ
0FT70ZZ
0FT74ZZ
0FT77ZZ
0FT78ZZ
0FT80ZZ
0FT84ZZ
0FT87ZZ
0FT88ZZ
0FQ90ZZ
0FQ93ZZ
0FQ94ZZ
0FQ97ZZ
0FQ98ZZ
0FQ90ZZ
0FQ93ZZ
0FQ94ZZ
0FQ97ZZ
0FQ98ZZ
0FR987Z
0FR98KZ
0FU987Z
0FU98JZ
0FU98KZ
0FQ50ZZ
0FQ53ZZ
0FQ54ZZ
0FQ57ZZ
0FQ58ZZ
0FQ60ZZ
0FQ63ZZ
0FQ64ZZ
0FQ67ZZ
0FQ68ZZ
0FQ70ZZ

0FQ73ZZ	0FQC8ZZ	0DQE7ZZ
0FQ74ZZ	0FRC87Z	0DQE8ZZ
0FQ77ZZ	0FRC8JZ	0FQ40ZZ
0FQ78ZZ	0FRC8KZ	0FQ40ZZ
0FQ80ZZ	0FUC87Z	0FQ40ZZ
0FQ83ZZ	0FUC8JZ	0FQ43ZZ
0FQ84ZZ	0FUC8KZ	0FQ43ZZ
0FQ87ZZ	0FQ40ZZ	0FQ43ZZ
0FQ88ZZ	0FQ43ZZ	0FQ44ZZ
0FR587Z	0FQ44ZZ	0FQ44ZZ
0FR58KZ	0FQ48ZZ	0FQ44ZZ
0FR687Z	0FQ40ZZ	0FQ48ZZ
0FR68KZ	0FQ43ZZ	0FQ48ZZ
0FR787Z	0FQ44ZZ	0FQ48ZZ
0FR78KZ	0FQ48ZZ	0FQ50ZZ
0FR887Z	0WQFXZ2	0FQ53ZZ
0FR88KZ	0DQ60ZZ	0FQ54ZZ
0FU587Z	0DQ60ZZ	0FQ57ZZ
0FU58JZ	0DQ63ZZ	0FQ58ZZ
0FU58KZ	0DQ63ZZ	0FQ60ZZ
0FU687Z	0DQ64ZZ	0FQ63ZZ
0FU68JZ	0DQ64ZZ	0FQ64ZZ
0FU68KZ	0DQ67ZZ	0FQ67ZZ
0FU787Z	0DQ67ZZ	0FQ68ZZ
0FU78JZ	0DQ68ZZ	0FQ70ZZ
0FU78KZ	0DQ68ZZ	0FQ73ZZ
0FU887Z	0DQ80ZZ	0FQ74ZZ
0FU88JZ	0DQ80ZZ	0FQ77ZZ
0FU88KZ	0DQ83ZZ	0FQ78ZZ
0F7C0DZ	0DQ83ZZ	0FQ80ZZ
0F7C0ZZ	0DQ84ZZ	0FQ83ZZ
0F7C3DZ	0DQ84ZZ	0FQ84ZZ
0F7C3ZZ	0DQ87ZZ	0FQ87ZZ
0F7C4DZ	0DQ87ZZ	0FQ88ZZ
0F7C4ZZ	0DQ88ZZ	0FQ90ZZ
0F7C7DZ	0DQ88ZZ	0FQ93ZZ
0F7C7ZZ	0DQ90ZZ	0FQ94ZZ
0F8G0ZZ	0DQ93ZZ	0FQ97ZZ
0F8G3ZZ	0DQ94ZZ	0FQ98ZZ
0FCC0ZZ	0DQ97ZZ	0FP40DZ
0FQC0ZZ	0DQ98ZZ	0FP43DZ
0FQC3ZZ	0DQA0ZZ	0FP44DZ
0FQC4ZZ	0DQA3ZZ	0FR50JZ
0FQC7ZZ	0DQA4ZZ	0FR54JZ
0FQC8ZZ	0DQA7ZZ	0FR58JZ
0FQC0ZZ	0DQA8ZZ	0FR60JZ
0FQC3ZZ	0DQE0ZZ	0FR64JZ
0FQC4ZZ	0DQE3ZZ	0FR68JZ
0FQC7ZZ	0DQE4ZZ	0FR70JZ

0FR74JZ	0FWD8YZ	0FBG4ZZ
0FR78JZ	0FWG0YZ	0FBG8ZZ
0FR80JZ	0FWG3YZ	0FBG0ZZ
0FR84JZ	0FWG4YZ	0FBG3ZZ
0FR88JZ	0F9G0ZX	0FBG4ZZ
0FR90JZ	0FBG0ZX	0FBG8ZZ
0FR94JZ	0F5D0ZZ	0DT90ZZ
0FR98JZ	0F5D3ZZ	0DT94ZZ
0FS40ZZ	0F5D7ZZ	0DT97ZZ
0FS44ZZ	0F5G0ZZ	0DT98ZZ
0F9D00Z	0F5G3ZZ	0FTG0ZZ
0F9D40Z	0FBD0ZZ	0FTG4ZZ
0F9D70Z	0FBD3ZZ	0D1607A
0F9G00Z	0FBD7ZZ	0D160JA
0F9G40Z	0FBG0ZZ	0D160KA
0F9G80Z	0FBG3ZZ	0D160ZA
0F9D0ZZ	0FTD0ZZ	0DT90ZZ
0F9D4ZZ	0FTD7ZZ	0DT90ZZ
0F9D7ZZ	0F9G0ZZ	0F190Z3
0F9G0ZZ	0F9G3ZZ	0F1G0ZC
0F9G4ZZ	0F9G4ZZ	0FTG0ZZ
0F9G8ZZ	0F9G8ZZ	0FTG0ZZ
0FCD0ZZ	0F1D0D3	0FYG0Z0
0FCD7ZZ	0F1D0DB	0FYG0Z1
0FCG0ZZ	0F1D0Z3	0FYG0Z2
0FCG3ZZ	0F1D0ZB	0FSG0ZZ
0FCG4ZZ	0F1D4D3	0FSG4ZZ
0FCG8ZZ	0F1D4DB	0FYG0Z0
0FFD0ZZ	0F1D4Z3	0FYG0Z1
0FFD3ZZ	0F1D4ZB	0FYG0Z2
0FFD4ZZ	0DB90ZZ	0F7D0DZ
0FFD7ZZ	0DB93ZZ	0F7D3DZ
0FFD8ZZ	0DB94ZZ	0F7D7DZ
0FHD0YZ	0DB97ZZ	0FHD0DZ
0FHD3YZ	0DB98ZZ	0FHD3DZ
0FHD4YZ	0FBG0ZZ	0FHD7DZ
0FHG0YZ	0FBG0ZZ	0FUD37Z
0FHG3YZ	0FBG3ZZ	0FUD47Z
0FHG4YZ	0FBG3ZZ	0FUD87Z
0FPD0YZ	0FBG4ZZ	0FQG0ZZ
0FPD3YZ	0FBG4ZZ	0FQG3ZZ
0FPD4YZ	0FBG8ZZ	0FQG4ZZ
0FPG0YZ	0FBG8ZZ	0FQG8ZZ
0FPG3YZ	0FBG0ZZ	0FRD87Z
0FPG4YZ	0FBG3ZZ	0FRD8JZ
0FWD0YZ	0FBG4ZZ	0FRD8KZ
0FWD3YZ	0FBG8ZZ	0FRF87Z
0FWD4YZ	0FBG0ZZ	0FRF8JZ
0FWD7YZ	0FBG3ZZ	0FRF8KZ

0FUD87Z
0FUD8JZ
0FUD8KZ
0FUF87Z
0FUF8JZ
0FUF8KZ
0F1D0D3
0F1D0DB
0F1D0Z3
0F1D0ZB
0F1D4D3
0F1D4DB
0F1D4Z3
0F1D4ZB
0F1G0D3
0F1G0DB
0F1G0Z3
0F1G0ZB
0F1G4D3
0F1G4DB
0F1G4Z3
0F1G4ZB
0F7D0ZZ
0F7D3ZZ
0FQD0ZZ
0FQD3ZZ
0FQD4ZZ
0FQD7ZZ
0FQD8ZZ
0DJ00ZZ
0DJ60ZZ
0DJD0ZZ
0DJU0ZZ
0DJW0ZZ
0WJG0ZZ
0WJJ0ZZ
0WJP0ZZ
0WJR0ZZ
0W3G0ZZ
0W3H0ZZ
0W3P0ZZ
0WJG0ZZ
0WJH0ZZ
0WJJ0ZZ
0D9U00Z
0D9U0ZZ
0D9V00Z
0D9V0ZZ
0D9W00Z
0D9W0ZZ
0W9G00Z
0W9G0ZZ
0WCJ0ZZ
0WCP0ZZ
0WCR0ZZ
0WJF4ZZ
0WJG4ZZ
0WJJ4ZZ
0WJP4ZZ
0WJR4ZZ
0D5U0ZZ
0D5U3ZZ
0D5U4ZZ
0D5V0ZZ
0D5V3ZZ
0D5V4ZZ
0D5W0ZZ
0D5W3ZZ
0D5W4ZZ
0DBU0ZZ
0DBU3ZZ
0DBU4ZZ
0DBV0ZZ
0DBV3ZZ
0DBV4ZZ
0DBW0ZZ
0DBW3ZZ
0DBW4ZZ
0DTU0ZZ
0DTU4ZZ
0WBH0ZZ
0WBH3ZZ
0WBH4ZZ
0DN84ZZ
0DNE4ZZ
0DNJ4ZZ
0DNU4ZZ
0DNV4ZZ
0DNW4ZZ
0FN04ZZ
0FN44ZZ
0FN48ZZ
0FN54ZZ
0FN64ZZ
0FN74ZZ
0FN84ZZ
0FN94ZZ
0FNG4ZZ
0FNG8ZZ
0DNE0ZZ
0DNE3ZZ
0DNJ0ZZ
0DNJ3ZZ
0DNU0ZZ
0DNU3ZZ
0DNV0ZZ
0DNV3ZZ
0DNW0ZZ
0DNW3ZZ
0FN00ZZ
0FN03ZZ
0FN40ZZ
0FN43ZZ
0FN50ZZ
0FN53ZZ
0FN57ZZ
0FN58ZZ
0FN60ZZ
0FN63ZZ
0FN67ZZ
0FN68ZZ
0FN70ZZ
0FN73ZZ
0FN77ZZ
0FN78ZZ
0FN80ZZ
0FN83ZZ
0FN87ZZ
0FN88ZZ
0FN90ZZ
0FN93ZZ
0FN97ZZ
0FN98ZZ
0FNG0ZZ
0FNG3ZZ
0DCU0ZZ
0DCU3ZZ
0DCU4ZZ
0DCV0ZZ
0DCV3ZZ
0DCV4ZZ
0DCW0ZZ
0DCW3ZZ
0DCW4ZZ
0WCG0ZZ
0WCG3ZZ
0WCG4ZZ

0W1G0J4
0W1G3J4
0W1G4J4
0W1G0JY
0W1G4JY
0W9J00Z
0W9J0ZZ
0W9J40Z
0W9J4ZZ
0WCH0ZZ
0WCH3ZZ
0WCH4ZZ
0WWG00Z
0WWG0JZ
0WWG30Z
0WWG3JZ
0WWG40Z
0WWG4JZ
