# Supplementary material for: Comparative Outcomes of Meropenem–Vaborbactam vs. Ceftazidime–Avibactam Among Adults Hospitalized with an Infectious Syndrome in the US, 2019–2021
Source: Antibiotics (Basel). 2025 Jan 3;14(1):29. doi: 10.3390/antibiotics14010029 (PMC11762528; doi:10.3390/antibiotics14010029)
Supplement: Supplementary file 1 [file antibiotics-14-00029-s001.zip › Supplemental Table S5.pdf]

Supplemental Table S5. Antimicrobials classification

1. Beta-lactams
    a. Carbapenem – ertapenem
    b. Antipseudomonal carbapenems – imipenem, meropenem, doripenem, meropenem-vaborbactam, imipenem-cilastatin-relebactam
    c. Penicillins – ampicillin, oxacillin
    d. Penicillins with beta-lactamase inhibitors – ampicillin-sulbactam
    e. Antipseudomonal penicillins with beta-lactamase inhibitors – piperacillin-tazobactam, ticarcillin-clavulanate
    f. Extended spectrum cephalosporins – cefotaxime, ceftriaxone, cefpodoxime, cefuroxime, cefdinir, cefiderocol
    g. Antipseudomonal cephalosporins – ceftazidime, cefepime, ceftolozane-tazobactam, ceftazidime-avibactam
    h. Monocyclic beta-lactam – aztreonam
2. Aminoglycosides – gentamicin, tobramicin, amikacin
3. Fluoroquinolones – ciprofloxacin, levofloxacin, moxifloxacin
    a. Respiratory fluorquinolones – levofloxacin, moxifloxacin
    b. Antipseudomonal fluoroquinolones – ciprofloxacin, levofloxacin
4. Folate pathway inhibitors – trimethoprim-sulfamothoxazole
5. Polymyxins – colistin (also called polymyxin E), polymyxin B
6. Tetracyclines – tetracycline, doxycycline, minocycline
7. Macrolides – erythromycin, azithromycin, clarithromycin
8. Glycopeptide – vancomycin
9. Oxazolidinone – linezolid
10. Glycycycline -- Tigecycline
