# Supplementary material for: Comparative Outcomes of Meropenem–Vaborbactam vs. Ceftazidime–Avibactam Among Adults Hospitalized with an Infectious Syndrome in the US, 2019–2021
Source: Antibiotics (Basel). 2025 Jan 3;14(1):29. doi: 10.3390/antibiotics14010029 (PMC11762528; doi:10.3390/antibiotics14010029)
Supplement: Supplementary file 1 [file antibiotics-14-00029-s001.zip › Supplemental Table S6.pdf]

Supplemental Table S6. Propensity Score Variables

Age
Male sex
Race
Hispanic
Admission Source
Surgical vs Medical
DNR code present on admission
Elixhauser Comorbidities
Congestive heart failure
Valvular disease
Pulmonary circulation disease
Peripheral vascular disease
Paralysis
Other neurological disorders
Chronic pulmonary disease
Diabetes without chronic complications
Diabetes with chronic complications
Hypothyroidism
Renal failure
Liver disease
Peptic ulcer disease with bleeding
AIDS
Lymphoma
Metastatic cancer
Solid tumor without metastasis
Rheumatoid arthritis/collagen vascular
Coagulopathy
Obesity
Weight loss
Fluid and electrolyte disorders
Chronic blood loss anemia
Deficiency anemia
Alcohol abuse
Drug abuse
Psychosis
Depression

Hypertension
Charlson Comorbidity Score
Hospital Characteristics
Census region
Midwest
Northeast
South
West
Number of Beds
<100
100 to 199
200 to 299
300 to 399
400 to 499
500+
Teaching Status
Urban vs Rural
Infection Type Category
Time to infection
Antibiotics within 90 days prior to admission
Severe sepsis
Septic shock
Illness severity measures prior to infection onset
Antibiotics administered by day 2 from infection onset
Beta-lactams
Carbapenems
Anti-pseudomonal carbapenems
Meropenem/vaborbactam
Imipenem/cilastatin/relebactam
Ertapenem
Penicillins
Penicillins with beta-lactamase inhibitors
Antipseudomonal penicillins with beta-lactamase inhibitors
Extended spectrum cephalosporins
Cefiderocol
Antipseudomonal cephalosporins
Ceftolozane/tazobactam
Ceftazidime/avibactam
Azrtreonam
Aminoglycosides

Fluoroquinolones
Respiratory quinolones
Antipseudomonal quinolones
Folate pathway inhibitors
Polymyxins
Tetracyclenes
Macrolides
Glycopeptide
Oxazolidinone
Glycycycline
