# Supplementary material for: Comparative Outcomes of Meropenem–Vaborbactam vs. Ceftazidime–Avibactam Among Adults Hospitalized with an Infectious Syndrome in the US, 2019–2021
Source: Antibiotics (Basel). 2025 Jan 3;14(1):29. doi: 10.3390/antibiotics14010029 (PMC11762528; doi:10.3390/antibiotics14010029)
Supplement: Supplementary file 1 [file antibiotics-14-00029-s001.zip › Supplemental Table S7.pdf]

Supplemental Table S7. Antibiotics administration

	MEV	%	CAZ	%	P-value
	N = 455		N = 2,320		
Antibiotics during hospitalization prior to index infection onset					
Beta-lactams	153	33.63%	746	32.16%	0.540
Carbapenems	12	2.64%	136	5.86%	0.683
Anti-pseudomonal carbapenems	7	1.54%	118	5.09%	0.001
Meropenem/vaborbactam	2	0.44%	1	0.04%	0.072
Imipenem/cilastatin/relebactam	2	0.44%	8	0.34%	0.673
Ertapenem	5	1.10%	31	1.34%	0.683
Penicillins	3	0.66%	13	0.56%	0.737
Penicillins with beta-lactamase inhibitors	3	0.66%	20	0.86%	1.000
Antipseudomonal penicillins with beta-lactamase inhibitors	45	9.89%	266	11.47%	0.330
Extended spectrum cephalosporins	58	12.75%	283	12.20%	0.744
Cefiderocol	0	0.00%	1	0.04%	1.000
Antipseudomonal cephalosporins	62	13.63%	212	9.14%	0.003
Ceftolozane/tazobactam	1	0.22%	1	0.04%	0.301
Ceftazidime/avibactam	8	1.76%	22	0.95%	0.127
Aztreonam	7	1.54%	16	0.69%	0.068
Aminoglycosides	5	1.10%	49	2.11%	0.153
Fluoroquinolones	16	3.52%	63	2.72%	0.348
Respiratory quinolones	12	2.64%	47	2.03%	0.408
Antipseudomonal quinolones	16	3.52%	63	2.72%	0.348
Folate pathway inhibitors	0	0.00%	2	0.09%	1.000
Polymyxins	2	0.44%	5	0.22%	0.323
Tetracyclenes	2	0.44%	17	0.73%	0.756
Macrolides	28	6.15%	101	4.35%	0.095
Glycopeptide	81	17.80%	381	16.42%	0.470
Oxazolidinone	5	1.10%	27	1.16%	0.976
Glycycycline	2	0.44%	4	0.17%	0.257
cIAI patients treated with metronidazole	2	0.44%	13	0.56%	0.270
Antibiotics administered by day 2 from infection onset					
Beta-lactams	445	97.80%	2,293	98.84%	0.079

Carbapenems	211	46.37%	604	26.03%	<0.001
Anti-pseudomonal carbapenems	201	44.18%	553	23.84%	<0.001
Meropenem/vaborbactam	139	30.55%	10	0.43%	<0.001
Imipenem/cilastatin/relebactam	1	0.22%	1	0.04%	0..301
Ertapenem	17	3.74%	62	2.67%	0.212
Penicillins	5	1.10%	19	0.82%	0.555
Penicillins with beta-lactamase inhibitors	8	1.76%	41	1.77%	0.989
Antipseudomonal penicillins with beta-lactamase inhibitors	127	27.91%	777	33.49%	0.020
Extended spectrum cephalosporins	82	18.02%	438	18.88%	0.668
Cefiderocol	0	0.00%	4	0.17%	1.000
Antipseudomonal cephalosporins	153	33.63%	1283	55.30%	<0.001
Ceftolozane/tazobactam	3	0.66%	21	0.91%	0.605
Ceftazidime/avibactam	12	2.64%	765	32.97%	<0.001
Aztreonam	13	2.86%	66	2.84%	0.988
Aminoglycosides	36	7.91%	180	7.76%	0.911
Fluoroquinolones	34	7.47%	173	7.46%	0.991
Respiratory quinolones	26	5.71%	133	5.73%	0.988
Antipseudomonal quinolones	34	7.47%	172	7.41%	0.965
Folate pathway inhibitors	0	0.00%	10	0.43%	0.383
Polymyxins	13	2.86%	58	2.50%	0.659
Tetracyclines	20	4.40%	82	3.53%	0.372
Macrolides	39	8.57%	205	8.84%	0.855
Glycopeptide	222	48.79%	1185	51.08%	0.372
Oxazolidinone	33	7.25%	147	6.34%	0.468
Glycylcycline	9	1.98%	23	0.99%	0.071
cIAI patients treated with metronidazole	3	0.66%	29	1.25%	0.346
Duration of antibiotics treatment course for index infection (days)					
Mean (SD)	7.0 (5.1)		7.1 (5.4)		0.637
Median [IQR]	5 [4, 8]		6 [4, 8]		0.954
Antibiotics during index hospitalization following completion of treatment for the index infection					
Beta-lactams	342	75.16%	1708	73.62%	0.493
Carbapenems	315	69.23%	916	39.48%	<0.001
Anti-pseudomonal carbapenems	311	68.35%	868	37.41%	<0.001

Meropenem/vaborbactam	305	67.03%	48	2.07%	<0.001
Imipenem/cilastatin/relebactam	23	5.05%	8	0.34%	<0.001
Ertapenem	23	5.05%	111	4.78%	0.806
Penicillins	12	2.64%	45	1.94%	0.337
Penicillins with beta-lactamase inhibitors	10	2.20%	71	3.06%	0.318
Antipseudomonal penicillins with beta-lactamase inhibitors	80	17.58%	476	20.52%	0.153
Extended spectrum cephalosporins	61	13.41%	248	10.69%	0.092
Cefiderocol	6	1.32%	29	1.25%	0.905
Antipseudomonal cephalosporins	144	31.65%	1565	67.46%	<0.001
Ceftolozane/tazobactam	19	4.18%	73	3.15%	0.178
Ceftazidime/avibactam	37	8.13%	1540	66.38%	<0.001
Aztreonam	9	1.98%	73	3.15%	0.178
Aminoglycosides	65	14.29%	372	16.03%	0.349
Fluoroquinolones	47	10.33%	291	12.54%	0.187
Respiratory quinolones	29	6.37%	189	8.15%	0.199
Antipseudomonal quinolones	47	10.33%	291	12.54%	0.187
Folate pathway inhibitors	9	1.98%	77	3.32%	0.131
Polymyxins	30	6.59%	120	5.17%	0.220
Tetracyclines	19	4.18%	99	4.27%	0.930
Macrolides	17	3.74%	52	2.24%	0.061
Glycopeptide	194	42.64%	941	40.56%	0.410
Oxazolidinone	70	15.38%	290	12.50%	0.094
Glycylcycline	31	6.81%	104	4.48%	0.035

MEV = meropenem/vaborbactam; CZA = ceftazidime/avibactam
Anti-pseudomonal carbapenems: doripenem, imipenem, meropenem
Definitions: Antibiotics used during the index hospitalization were divided into three categories: 1) antibiotics administered prior to infection onset (e.g., antibiotics during hospitalization prior to index infection onset); 2) antibiotics which constitute early therapy during an infectious syndrome episode (e.g., antibiotics administered by day 2 from infection onset); and, 3) antibiotics which were administered at any time following resolution of infection (e.g., antibiotics received during hospitalization following treatment of the infection onset).
