# Supplementary material for: Comparative Outcomes of Meropenem–Vaborbactam vs. Ceftazidime–Avibactam Among Adults Hospitalized with an Infectious Syndrome in the US, 2019–2021
Source: Antibiotics (Basel). 2025 Jan 3;14(1):29. doi: 10.3390/antibiotics14010029 (PMC11762528; doi:10.3390/antibiotics14010029)
Supplement: Supplementary file 1 [file antibiotics-14-00029-s001.zip › Supplemental Table S8.pdf]

Supplemental Table S8. Elixhauser comorbidities

	Mer/Vab	%	Cef/Avi	%	P-value
	N = 455		N = 2320		
Elixhauser Comorbidities					
Congestive heart failure	149	32.75%	735	31.68%	0.655
Valvular disease	50	10.99%	235	10.13%	0.581
Pulmonary circulation disease	51	11.21%	275	11.85%	0.696
Peripheral vascular disease	56	12.31%	289	12.46%	0.930
Paralysis	44	9.67%	282	12.16%	0.132
Other neurological disorders	208	45.71%	1131	48.75%	0.236
Chronic pulmonary disease	115	25.27%	720	31.03%	0.014
Diabetes without chronic complications	26	5.71%	207	8.92%	0.024
Diabetes with chronic complications	200	43.96%	884	38.10%	0.019
Hypothyroidism	85	18.68%	375	16.16%	0.187
Renal failure	184	40.44%	835	35.99%	0.072
Liver disease	95	20.88%	384	16.55%	0.026
Peptic ulcer disease with bleeding	8	1.76%	35	1.51%	0.693
AIDS	2	0.44%	19	0.82%	0.559
Lymphoma	5	1.10%	33	1.42%	0.587
Metastatic cancer	18	3.96%	88	3.79%	0.868
Solid tumor without metastasis	35	7.69%	164	7.07%	0.637
Rheumatoid arthritis/collagen vascular	21	4.62%	106	4.57%	0.965
Coagulopathy	141	30.99%	706	30.43%	0.813
Obesity	145	31.87%	641	27.63%	0.067
Weight loss	158	34.73%	845	36.42%	0.491
Fluid and electrolyte disorders	375	82.42%	1856	80.00%	0.235
Chronic blood loss anemia	6	1.32%	45	1.94%	0.367
Deficiency anemia	43	9.45%	272	11.72%	0.162
Alcohol abuse	32	7.03%	126	5.43%	0.178
Drug abuse	25	5.49%	120	5.17%	0.778
Psychosis	4	0.88%	58	2.50%	0.035
Depression	80	17.58%	453	19.53%	0.336
Hypertension	344	75.60%	1636	70.52%	0.028
